# Case of Acne Rosacea

**Published:** 1848-03

**Authors:** A. O. Kellog

**Affiliations:** Mariposa, C. W.


ART. II.—*Case of Acne Rosacea. Treated with Liquor Potassae Arsenitis.* Reported by A. O. Kellog, M.D., of Mariposa, C.W.

John McFaggart, aged fifty, farmer, of good general health, had been afflicted for nearly four years with Acne Rosacea. He had taken medicine of almost every description during that time, without any apparent benefit. He first consulted me, July 6th, 1847, and at that time was applying what appeared to me to be a solution of the bi-chloride of mercury, and taking sulphur, &c., prescribed by an apothecary. The following was his condition at the time :— The alimentary canal was extremely irritable ; the only diet the patient could bear consisted of milk, rice, toast, &c. Every thing of an oily nature brought on diarrhœa, and aggravated the eruption. This occupied the whole of the face, extending into the hair above the forehead. There were also patches on the fore arms, and groin. The sub-cutaneous cellular tissue seemed deeply involved, indurated, giving the skin a rough, hard, uneven feeling to the hand passed over it. The projecting pustules were of a reddish tint, inclining to a brown shade. Some of them discharged a bloody purulent matter, forming slight incrustations ; others discharged only when pressed. There was no itching exeept on approaching the fire, while warm in bed, or when he took sufficient exercise to produce perspiration ; at these times he expressed it as intolerable.

I gave a guarded prognosis, explained to the patient the difficulty of cure by ordinary means, and he consented to follow patiently for a year any course of treatment which might possibly succeed in relieving him of his disgusting malady.

With this assurance, I determined to make a fair trial of the arsenic, but not having any by me at the time, I prescribed the comp. decoction of sarsaparilla with iodine, and told him to return in a few weeks. July 27, no change in the eruption. Notwithstanding the irritability of the alimentary canal, I now determined to commence with the arsenic.

I had prepared the medicine myself, according to the United States' Pharmacopœa, and directed him to take five drops of the liquor-potassae-arsenitis, three times a day with his meals, till a slight conjunctivitis ensued, when he was instructed to reduce the dose to three or four drops. August 8th, the medicine has had the desired effect. There is a slight conjunctivitis with lachymation ; otherwise, he feels no effect from the medicine whatever. The itching continues as usual. The eruption on the face appears to be nearly, if not quite, as bad as ever. It is better, however, on the arms and groin. To continue the medicine.

Sept. 25th.—Has taken the medicine regularly. The disease appears to be, as he expresses it, "at a stand-still," yet the eruption on the arms and groin has nearly disappeared ; the pustules on the face are paler ; no discharge except on pressure. To continue the medicine—take four drops as usual. Oct. 27—visited me again. The disease he yet thinks "at a stand-still ;" itching, on approaching the fire, or taking exercise, as great as ever on the face ; no eruption on the arms or groin. The medicine to be continued.

Nov. 24th.—Visited me again. Says he thinks the medicine has "lost its strength, as it does not *affect his eyes*, as formerly." The eruption on the face, however, is better ; some itching, as usual. I now directed him to take seven drops three times a day, with his meals. This dose he continued to take for a month, when he visited me again, to let me know, as he said, that he was *cured*. The eruption has quite disappeared, except a few small pustules on the forehead near the edge of the hair ; no itching, whatever ; skin smooth. He said he felt a decided change for the better directly after he began to take seven drops at a dose. I directed him to continue, lest the old enemy should re-appear, and should it, after discontinuing the medicine altogether. I have no doubt of being able to meet and conquer it with the same weapon. During the six months the patient was under treatment, he must have taken from three to four ounces of the liquor-potassae-arsenitis, without any one bad symptom whatever, notwithstanding the extreme irritability of the alimentary canal when he commenced its use. This attribute to the precaution of taking it on a full stomach. Administered in this way, it is not only free from all those bad effects spoken of by authors, but calculated to *remove* some of the very symptoms, (irritation of the alimentary canal, &c., which it is said to *bring on*, as in the case of the patient above), for which we are told to discontinue it altogether, and thus deprive ourselves of a valuable remedy, for which, in certain forms of skin disease, we have no substitute in the whole catalogue of medicines. No other remedy whatever was taken by the patient during the time he was taking the arsenic, except it might be occasionally a mild laxative. This shows that it is alone sufficient for the cure of a class of skin diseases which have been regarded by high authority as incurable. How it acts we cannot say—hardly form a theory. The fact of its efficacy, however, is certain, and this is sufficient. I have other cases which I am treating with the same medicine. One of *Lupus*, the result of which you shall be apprized of. For further information on this interesting subject, I would refer the reader to Mr. Hunt's papers in the Lancet, 1846, and to the interesting case of Dr. Chambers, in the same journal, and the same condensed in the fourteenth number of Braithwaite's Retrospect.

